# Minimax and admissible adaptive two-stage designs in phase II clinical trials

**DOI:** 10.1186/s12874-016-0194-3

**Published:** 2016-08-02

**Authors:** Guogen Shan, Hua Zhang, Tao Jiang

**Affiliations:** 1 Epidemiology and Biostatistics Program, Department of Environmental and Occupational Health, School of Community Health Sciences, University of Nevada Las Vegas, Las Vegas, 89154 NV USA; 2 School of Computer and Information Engineering, Zhejiang Gongshang University, Hangzhou310018 Zhejiang, China; 3 Department of Statistics, Zhejiang Gongshang University, HangzhouZhejiang, 310018 China

**Keywords:** Adaptive design, Admissible design, Efficacy, Futility, Minimax design, Simon’s design

## Abstract

**Background:**

Simon’s two-stage design is the most widely implemented among multi-stage designs in phase II clinical trials to assess the activity of a new treatment in a single-arm study. In this two-stage design, the sample size from the second stage is fixed regardless of the number of responses observed in the first stage.

**Methods:**

We develop a new minimax adaptive design for phase II clinical trials, by using the branch-and-bound intelligent algorithm based on conditional error functions.

**Results:**

We compare the performance of the proposed design and competitors, including Simon’s minimax design, and a modified Simon’s design that allows early stopping for futility or efficacy. The maximum sample size of the proposed minimax adaptive design is guaranteed to be less than or equal to those from other existing designs. When the proposed design has the same maximum sample size as others, it always has the smallest expected sample size. In addition to the minimax adaptive design, we also introduce admissible adaptive designs determined from a Bayesian perspective.

**Conclusions:**

The proposed adaptive minimax design can save sample sizes for a clinical trial. The minimum required sample size is critical to reduce the cost of a project.

## Background

In phase II clinical trials, a new treatment or a new therapy is often assessed by measuring activity with dichotomized endpoints, responding ’yes’ or ’no’ to the intervention. For Oncology clinical trials, the response criteria may be determined by the Response Evaluation Criteria In Solid Tumours (RECIST) [1]. The traditional experiment in phase II Oncology trials is often conducted in a single arm study, which is also popular in other studies, such as AIDS. All patients enrolled in the study are treated with the same treatment, and their measurements are obtained at the end of the study and compared to the priori estimate from historical studies with the similar condition of experiment and patients. From ethical and economical considerations, a trial should be allowed to stop earlier after an interim analysis to better protect patients, especially in situations when the treatment is indeed ineffective. For this reason, a multi-stage design is often implemented, and among these designs the most popular is Simon’s two-stage design [2]. Simon [2] proposed two optimal designs: the optimal design with the expected sample size under the null (ESS_0_) minimized, and the minimax design having the smallest ESS_0_ among the designs with the maximum sample size (MSS) minimized. Simon’s design allows early stopping in the first stage for futility only. Recently, Mander and Thompson [3] extended Simon’s design to allow stopping for efficacy or futility by introducing an additional design parameter that represents the stopping criteria for efficacy in the first stage. It is guaranteed that the MSS of the modified design is less than or equal to that of Simon’s.

In Simon’s design and the modified Simon’s design due to Mander and Thompson [3], the second stage sample size is always fixed and is not allowed to be modified as the result observed from the first stage. To make a design flexible and efficient, adaptive designs have been developed to allow the second stage sample size to depend on first stage responders. It is easy to show that Simon’s optimal design is a special case of adaptive designs, therefore, the expected sample size of an optimal adaptive design is always less than or equal to that of Simon’s design. Several optimal adaptive designs have been developed for phase II clinical trials, and the majority of them are based on the optimal criteria with the smallest ESS_0_. Banerjee and Tsiatis [4] developed an optimal adaptive two-stage design by using a Bayesian decision-theoretic construct to minimize the expected loss through backward induction, with type I and II error rates respected. The sample size savings are small to modest when compared to Simon’s optimal design. Later, Englert and Kieser [5] developed an optimal adaptive two-stage design based on conditional error functions [6] and an efficient search strategy [7]. Although these adaptive designs guarantee the type I and II error rates, these designs suffer from a counter-intuitive feature that the second stage sample size may increase as the number of responses observed from the first stage increases. Very recently, Shan et al. [8] developed an optimal adaptive two-stage design with the monotonicity property respected; the second stage sample size is a non-increasing function of the first stage responders. This improvement makes it practical to use the optimal adaptive design.

In phase II clinical trials, it is desirable to achieve the primary goal of the study with the number of patients minimized, as the cost of the study highly depends on the number of patients. In addition, Institutional Review Boards approve proposed studies based on the maximum possible number of patients that are needed to address the scientific questions. Therefore, the minimax design is preferable by researchers with the smallest MSS as compared to the optimal design when the MSS difference between the two designs is not small. To our best knowledge, no adaptive design based on the minimax criteria has been proposed for use in practice. Due to the importance of such designs, we develop a new minimax adaptive design with the monotonic property respected in this article by using the branch-and-bound algorithm [7] based on conditional type I and II error rates.

Both minimax and optimal designs have been widely used in clinical trials. It is often the case that the expected sample size of the minimax design is much larger than that of the optimal design, although the minimax design has a smaller maximum sample size. To compromise between the maximum sample size and the expected sample size under the null, an admissible adaptive design was proposed by Jung et al. [9], which was implemented in Java language by them. By using the Bayes risk function as in Jung et al. [9], we propose a new admissible adaptive two-stage design that is between the minimax adaptive design and the optimal adaptive design.

The remainder of this article is organized as follows. In “Methods” Section, we introduce the detailed search method for the optimal adaptive design when the first stage and the MSS of the second stage sample size are fixed, then present the approach to find the minimax adaptive design. In “Results” Section we compare the MSS and the ESS_0_ of the proposed minimax adaptive design with competitors. A real clinical trial from a cancer study is used to illustrate the proposed design at the end of “Results” Section. Finally, we provide some remarks in “Discussion and conclusions” Section.

## Methods

Simon [2] proposed the widely used two-stage designs for early phase II clinical trials with binary endpoints by testing the response rate to make a conclusion of go or no-go to the next trial phase of the study. In this study design, the unacceptable response rate *π*_*u*_ can be estimated from historical data, and the acceptable response rate *π*_*a*_ is often the targeted response rate of a new treatment or therapy, where *π*_*u*_<*π*_*a*_. For example, in the clinical trial for urothelial cancer with neoadjuvant therapy [10], the unacceptable and acceptable response rates are *π*_*u*_=35 % and *π*_*a*_=50 %, respectively. The hypotheses to be tested are

$$H_{0}: \pi\leq \pi_{u}, $$

against

$$H_{a}: \pi\geq \pi_{a}. $$

The null hypothesis is rejected for a large response rate. Let *n*_1_, *n*_2_, and *n* be the number of subjects enrolled in the first stage, the second stage, and both stages combined, respectively, and *x*_1_, *x*_2_, and *x* are the associated number of responses observed from the study.

In the clinical trial of the neoadjuvant therapy for urothelial cancer [10], Simon’s minimax design was used for sample size determination to achieve 80 % power (*β*=0.2) at the significance level of *α*=0.1 when the response rates were *π*_*u*_=35 % and *π*_*a*_=50 %. The design was calculated as: (*r*_1_/*n*_1_,*r*/*n*)=(10/31,21/49) with the ESS_0_=40.8. The trial was allowed to stop for futility at the first stage if the number of first stage responses *x*_1_≤10 was observed from a total of *n*_1_=31 patients. Otherwise, an additional *n*_2_=*n*−*n*_1_=49−31=18 patients were enrolled in the second stage, and at least 22 responses should be observed from total 49 patients, *x*≥22, in order to claim that the neoadjuvant therapy had sufficient activity. The MSS of the minimax design was 49. An alterntive to the minimax design is Simon’s optimal design whose ESS_0_ was the smallest among all designs that met the design criteria. The design parameters for the optimal design are: (*r*_1_/*n*_1_,*r*/*n*)=(7/20,24/58) with the ESS _0_=35.2. As expected, the ESS_0_ of the optimal design is less than that of the minimax design (35.2 agaisnt 40.8), but the MSS is much larger for the optimal design as compared to the minimax design (58 against 49).

A modified Simon’s design that allows early stopping for futility or efficacy, was proposed by Mander and Thompson [3] who introduced another design parameter, *r*_2_, as ((*r*_1_,*r*_2_)/*n*_1_,*r*/*n*). This design is referred to as the Minimax-EF design. For the aforementioned cancer study, the design can be calculated by using the Stata package, *simon*2*stage* [3], as ((*r*_1_,*r*_2_)/*n*_1_,*r*/*n*)=((11,16)/32,21/49), see Table 1. With *n*_1_=32 patients enrolled in the first stage of the study, the study will be stopped for futility if *x*_1_≤11 or efficacy if *x*_1_>16. A decision can not be made in the first stage when 11<*x*_1_≤16, and additionally *n*_2_=*n*−*n*_1_=49−32=17 patients will be enrolled in the second stage. At the end of the study, the null hypothesis will be rejected if *x*>21. Otherwise, it is concluded that the new treatment is not promising enough to warrant further investigation. The ESS_0_ of the minimax-EF design is 39.2 as compared to the ESS _0_=40.8 from Simon’s minimax design.

**Table 1:** The proposed adaptive minimax design for the urothelial cancer trial with the neoadjuvant therapy with (*α*,*β*,*π*_*u*_,*π*_*a*_)=(0.1,0.2,0.35,0.5)

S	*n*_2_(*S*)	*n*(*S*)	*r*(*S*)
Minimax-EF design			
≤11	0	32	0
12	17	49	21
13	17	49	21
14	17	49	21
15	17	49	21
16	17	49	21
≥17	0	32	0
Minimax adaptive design			
≤9	0	28	0
10	21	49	21
11	21	49	21
12	21	49	21
13	21	49	21
14	19	47	20
15	18	46	20
≥16	0	28	0

In the two aforementioned designs, the second stage sample size is not allowed to change with the number of responses observed from the first stage. To improve the efficiency and flexibility of a study, we propose a new adaptive two-stage design based on the minimax criteria from Simon’s, the design with the smallest ESS_0_ and MSS. In the proposed adaptive design, the second stage sample size, *n*_2_(*S*), depends on the number of first stage responses, *S*, and *n*_2_(*S*) is a non-increasing function of *S*, specifically, *n*_2_(*S*_1_)≥*n*_2_(*S*_2_) when *S*_1_<*S*_2_. For a given first stage sample size *n*_1_, the value of *S* ranges from 0 to *n*_1_: *S*=0,1,…,*n*_1_. Moreover, the associated critical value for each *S*, *r*(*S*), also needs to be determined for the adaptive design. Then, the proposed design is presented as

$$n_{1}\ \text{and} ~\left(n_{2}(S),r(S)\right),~ S=0,1,2,\cdots,n_{1}, $$

with a total of 2*n*_1_+3 unknown parameters. As pointed out by many researchers [5, 8], it becomes quickly impossible to estimate these parameters by enumerating all attainable values of *n*_2_(*S*) and *r*(*S*), even after controlling for the upper bound of the second stage sample size.

Conditional error functions are frequently used in adaptive designs to fully use the information from the previous stage, specifically,

$$P(s|r(s),n_{2}(s),\pi)=1-B\left(r(s)-s:n_{2}(s),\pi\right), $$

where *s* is the observed first stage response under the design parameters *n*_2_(*s*) and *r*(*s*), and *B*(*x*:*y*,*z*) is the cumulative probability function of a binomial distribution for observed value *x* with size *y* and probability *z*. Note that *P*(*s*|*π*)=0 or 1 when the study is terminated after the first stage for futility or efficacy, respectively. As a special case, it is always reasonable to stop the trial when no response is observed from the first stage, *P*(0|*π*)=0. It should be noted that the proposed design allows early stopping in the first stage for either futility or efficacy just as existing adaptive designs.

For each design, the type I and II error rates are then calculated from conditional error functions as

$$\alpha=\sum\limits_{s=0}^{n_{1}}P(s|r(s), n_{2}(s), \pi_{u})\times b(s:n_{1},\pi_{u}), $$

and

$$\beta=1-\sum\limits_{s=0}^{n_{1}}P(s|r(s), n_{2}(s), \pi_{a})\times b(s:n_{1},\pi_{a}), $$

where *b*(.) is the density function of a binomial distribution. All designs with guaranteed type I and II error rates, are candidates for the optimal design. Often, multiple designs meet the design criteria, and an additional criteria should be applied in order to find the optimal design. The criteria used in the proposed adaptive minimax design is the smallest ESS_0_ and MSS,

1 $$ \min_{\max(n_{1}+n_{2}(s),s=0,1,\ldots,n_{1})}ESS_{0},  $$

where ESS$_{0}=\sum _{s=0}^{n_{1}}[n_{1}+n_{2}(s)]\times b(s:n_{1},\pi _{u})$ is the expected sample size under the null for the design with *n*_1_ and max(*n*_1_+*n*_2_(*s*),*s*=0,1,…,*n*_1_) as the first stage sample size and the MSS. The min in Eq. (1) is used in two folders. The function is first used to find all satisfied designs with the smallest MSS, $\min _{\max (n_{1}+n_{2}(s),s=0,1,\ldots,n_{1})}$. The second is to identify the minimax adaptive design as the one from these in the previous step with the smallest ESS_0_.

We start the design search with a fixed first stage sample size *n*_1_ and the MSS *n*. Then, the MSS of the second stage is *n*_2,*max*_=*n*−*n*_1_. It is easy to show that *n*_2_(*S*)≤*n*_2,*max*_ and *r*(*S*)≤*n*_2_(*S*). The optimal design needs to be searched over a triangle space for each *S*, *ϕ*(*S*)={(*n*_2_(*S*),*r*(*S*)):*r*(*S*)≤*n*_2_(*S*)≤*n*_2,*max*_}, where *S*=0,1,2,⋯,*n*_1_. The complete search space is a product of these triangle spaces,

$$\left(\phi(0)\times \phi(1)\times \cdots \times \phi(n_{1})\right). $$

As the first stage sample size *n*_1_ increases, the size of this complete search space increases exponentially. Therefore, it is not feasible to conduct this naive search to identify the optimal design.

It is much more complicated to search for an optimal solution over a two-dimensional space than a one-dimensional space. For this reason, Englert and Kieser [5] suggested using the union of all type I conditional error functions and (0,1), referred to as *Ω*, as the parameter space. For each element in *Ω*, it contains the information of *n*_2_(*S*) and *r*(*S*) as in the two-dimensional space. That said, it is equivalent to determine the conditional type I error value for *S* and (*r*(*S*),*n*_2_(*S*)). It is still not feasible to conduct a grid search over the parameter space (*a*(*n*_1_+1)−dimensional space) due to the fact that the size of the parameter space increases very quickly as *n*_1_ and *n*_2,*max*_ go up.

In order to overcome the computational burden, the branch-and-bound algorithm [7], an intelligent algorithm, is considered when searching for the optimal design over a one-dimensional space on each *S*. This algorithm can be used to search for the optimal design with or without constraints [5, 8]. The monotonicity restriction in the optimal adaptive design search by Shan et al. [8] is an important feature that makes a design usable in practice. The second stage sample size is a non-increasing function of the number of responses observed from the first stage: *n*_2_(*S*_1_)≥*n*_2_(*S*_2_) when *S*_1_<*S*_2_. This monotonicity restriction is respected in the proposed minimax adaptive design.

As pointed out, it is time consuming to compute the actual type I and II error rates for each element in the parameter space, and the branch-and-bound algorithm is able to finish the design search in a timely manner by discarding elements that do not lead to the optimal design, which is the key idea of this intelligent algorithm. When the sample sizes (*n*_1_,*n*) are given, the ESS_0_ is the objective function. Two procedures are recursively utilized in the algorithm to identify the optimal design. The first procedure is the branching process that splits the problem into several complement problems. The conditional type I error functions are used in this step to split problems. Although it is not a requirement to sort the elements in *Ω* in the design search, it helps to reduce the computational intensity to sort them by *n*_2_(*S*) in an ascending order, and *P*(*S*|*π*_*u*_) in an increasing order. The ordering of *n*_2_(*S*) is used to meet the monotonicity feature of the proposed design.

The second procedure, the bounding procedure, computes boundary values of constraint functions. Let *O*(*S*,*W*_*S*_) be the *W*_*S*_-th conditional error function in the *Ω* when the number of responses is *S* in the first stage. Suppose the current branching outcome from the branching procedure is at *S*=*k*

2 $$ {\small{\begin{aligned} {}O(0,1), \!O(\!1,W_{1}), \!O(2,\!W_{2}), \ldots,\! O(k,\!W_{k}), O(k\,+\,\!1,\!1),\ldots,\!O(n_{1},\!1). \end{aligned}}}  $$

When no response is observed from the first stage, the trial is assumed to be stopped for futility, that is represented by *O*(0,1). Note that we use *n*_2_(*S*,*W*_*S*_) and *r*(*S*,*W*_*S*_) to replace *n*_2_(*S*) and *r*(*S*) in the design search. The objective function at the current branching step is calculated as

$${}f\,=\,\sum\limits_{s=0}^{k}\left(n_{1}+n_{2}(s,w_{s})\right)\times b(s:n_{1},\pi_{u}) +\! \sum\limits_{s=k+1}^{n_{1}} \!n_{1} b(s:n_{1},\pi_{u}). $$

The overall goal is to find the values of *W* for each *S* in *O*(*S*,*W*) that minimizes the objective function as

$$\min_{O(S, W_{S}),S=0,1,\ldots,n_{1}} f. $$

Two constraints need to be satisfied in the design search

$$\alpha_{min}=\sum\limits_{s=0}^{k}P\left(s|r(s,w_{s}),n_{2}(s,w_{s}),\pi_{u}\right)\times b(s:n_{1},\pi_{u}), $$

and

$$ \begin{aligned} {}\beta_{min}&=1-\sum\limits_{s=0}^{k}P\left(s|r(s,w_{s}),n_{2}(s,w_{s}),\pi_{a}\right)\times b(s:n_{1},\pi_{a})\\  &\quad - \sum\limits_{s=k+1}^{n_{1}} b(s:n_{1},\pi_{a}). \end{aligned}  $$

These two constraints help to determine the set of feasible solutions, and discard the candidates that do not lead to the optimal design.

The minimax-EF design is a special case of the minimax adaptive design, therefore, the MSS of the minimax-EF design is the upper bound of the proposed adaptive design. For this reason, we start the search with the MSS, *n*_*t*_, which is the MSS of the minimax-EF design. For this given MSS, say *n*_*t*_, the possible number of subjects from the first stage, *n*_1_, ranges between 1 and *n*_*t*_−1. The search for *n*_1_=1 and *n*_*t*_−1 as the first stage sample sizes are excluded for practical reasons: it is not realistic to enroll only one patient to make a decision.

For each sample size configuration (*n*_1_,*n*_*t*_), the algorithm is applied for the design search. If the study is stopped for futility when *S*≤*s*−1, then we assign *n*_2_(*s*,*W*_*s*_)=*n*−*n*_1_ to guarantee that the MSS is exactly *n*_*t*_. It should be noted that the MSS could occur at multiple *S* values. The ascending order of *n*_2_(*S*) for elements in parameter space *Ω*, is useful to meet the monotonic relationship between the *n*_2_(*S*) and *S* in searching for the design. Among these obtained optimal adaptive designs, the one with the smallest ESS_0_ is the adaptive minimax design when the MSS is *n*_*t*_. From the relationship between the proposed design and the minimax-EF design, it is guaranteed that an optimal adaptive design will be obtained when MSS is *n*_*t*_. The MSS is then decreased by 1, and the optimal adaptive design is searched again with the MSS= *n*_*t*_−1. This procedure will be continued until no optimal design is obtained from three consecutive MMS values, say *n*^∗^−1, *n*^∗^−2, and *n*^∗^−3. Then, *n*^∗^ is the minimum MSS, and the optimal design associated with *n*^∗^ is the final minimax adaptive two-stage design. It is obvious that *n*^∗^≤*n*_*t*_.

For the candidates of an admissible design, the first step is to identify the MSS values of the minimax adaptive design and the optimal adaptive design by Shan et al. [8], *n*_*min*_ and *n*_*opt*_ that are in the range of the MSS of an admissible design. Secondly, for each given MSS, *n*, between *n*_*min*_ and *n*_*opt*_, the optimal design with the smallest ESS_0_ is calculated by using the algorithm aforementioned. The sample size information, *n* and ESS_0_, are used in calculating the Bayes risk function

$$T=q\times n+(1-q)\times ESS_{0}=(n-ESS_{0})q+ESS_{0}, $$

where *q* is a pre-specified weight value, *q*∈[0,1]. It can be seen that the Bayes risk function *T* is a linear function of *q* with *n*−*ESS*_0_ as the slope and *ESS*_0_ as the intercept. As *ESS*_0_ is always less than *n*, *T* is an increasing function of *q*.

## Results

We compare performance of the proposed minimax adaptive design, Simon’s minimax design, the minimax-EF design, and the optimal adaptive design due to Shan et al. [8]. The first three designs are minimax designs, while the last one is under the optimal criteria. The first design and the last design are adaptive designs. To the best of our knowledge, we do not find a direct competitor in the category of adaptive two-stage designs under the minimax criteria. Simon’s minimax design is the most commonly used design under the minimax criteria, thus it is included in the comparison. The minimax-EF design stops for either futility or efficacy in the first stage. This stopping rule is also applied in the proposed design, thus, this design is also included in the comparison. Simon’s design only allows stopping of the trial at the first stage for futility, and the other three designs allows the stoppage for either futility or efficacy in the first stage.

The MSS and the ESS_0_ are compared in Table 2 for the proposed minimax adaptive design, and the other three competitors when *π*_*a*_−*π*_*u*_=0.2, and 0.15 at the significance level of *α*=0.05. As expected, the proposed minimax adaptive design has a smaller or the same MSS as compared to the Simon’s minimax design and the minimax-EF design. When the proposed design has the same MSS as either of the two minimax designs, the ESS_0_ of the proposed design is always smaller. For example, the saving of the ESS_0_ from the proposed design as compared to the minimax-EF design when they have the same MSS in Table 2, ranges from 0.03 to 10.16, with an average of 3.07 patients. The optimal adaptive design is included as a reference, and the MSS of this design is generally larger than that of the proposed minimax adaptive design, with a range from 2 to 16, and an average of 9.3 patients from all the configurations studied in Table 2.

**Table 2:** Comparison between three optimal designs for expected sample size *E* *S* *S*_0_ at *α*=0.05 given *π*_*a*_−*π*_*u*_=0.2 and 0.15

			Minimax						Optimal	
			Simon		Minimax-EF		Adaptive		Adaptive	
*π*_*u*_	*π*_*a*_	Power	*n*	*E* *S* *S*_0_	*n*	*E* *S* *S*_0_	*n*	*E* *S* *S*_0_	*n*	*E* *S* *S*_0_
*π*_*a*_−*π*_*u*_=20 %										
0.1	0.3	0.8	25	19.51	24	20.30	23	20.94	29	14.85
		0.9	33	26.18	33	23.96	33	23.93	35	22.38
0.2	0.4	0.8	33	22.25	32	24.93	32	23.22	37	20.48
		0.9	45	31.23	44	35.68	44	33.39	53	29.74
0.3	0.5	0.8	39	25.69	36	30.68	36	29.31	46	23.45
		0.9	53	36.62	50	42.47	50	41.03	60	34.08
0.4	0.6	0.8	39	34.44	39	34.33	39	26.86	46	24.39
		0.9	54	38.06	54	38.03	53	42.65	66	35.64
0.5	0.7	0.8	37	27.74	37	26.90	37	26.87	43	23.33
		0.9	53	36.11	51	41.14	51	37.74	59	33.45
0.6	0.8	0.8	35	20.77	33	23.97	33	22.13	38	20.28
		0.9	45	35.90	45	33.30	45	31.36	52	28.74
0.7	0.9	0.8	26	23.16	26	23.11	25	18.00	27	14.82
		0.9	32	22.66	32	22.66	32	22.64	36	20.80
*π*_*a*_−*π*_*u*_=15 %										
0.1	0.25	0.8	40	28.84	38	33.94	38	28.87	43	24.49
		0.9	55	40.03	53	47.87	53	41.29	62	36.45
0.2	0.35	0.8	53	40.44	53	40.41	53	40.33	63	34.87
		0.9	77	58.42	76	66.51	74	59.58	87	50.80
0.3	0.45	0.8	65	49.63	64	51.32	64	48.08	77	41.33
		0.9	88	78.51	88	78.45	88	68.29	104	59.96
0.4	0.55	0.8	70	60.07	69	54.17	69	49.84	82	44.05
		0.9	94	78.88	94	76.30	94	74.20	106	63.84
0.5	0.65	0.8	68	66.11	68	66.05	67	58.41	81	43.01
		0.9	93	75.00	93	72.20	93	69.84	109	61.87
0.6	0.75	0.8	62	43.79	62	42.89	61	45.26	69	38.53
		0.9	84	73.20	84	73.13	84	64.00	97	54.99
0.7	0.85	0.8	49	34.44	49	34.36	49	33.00	59	29.78
		0.9	68	48.52	65	50.46	65	48.78	78	42.60

**Fig. 1:**
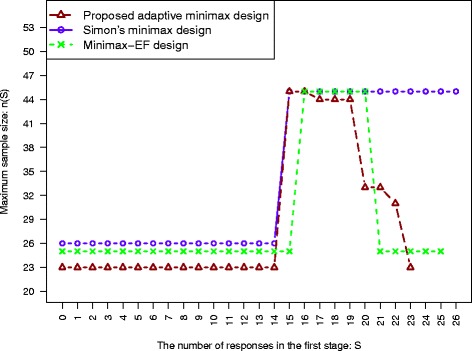
The comparison among Simon’s minimax design, the minimax-EF design and the proposed adaptive minimax design for the design with parameters (*α*,*β*,*π*_*u*_,*π*_*a*_)=(0.05,0.1,0.6,0.8). The maximum sample size, *n*(*S*), is plotted as a function of the number of responses from the first stage, *S*

We present the proposed minimax adaptive design with specific design parameters in Tables 3 and 4 for *π*_*a*_=*π*_*u*_+0.2 and *π*_*a*_=*π*_*u*_+0.15, respectively. The pre-specified type I error rate is set as *α*=0.05, and two type II error rates are studied, *β*=0.1 and 0.2. We present the minimum adaptive design for various values of *π*_*u*_, from 0.2 to 0.7. For example, for the design to achieve 90 % power with *π*_*u*_=0.6, *π*_*a*_=0.8 as in Table 3, Simon’s minimax design, the minimax-EF design and the proposed adaptive minimax design are displayed in Fig. 1: the MSS of the study (*n*(*S*)) VS the number of responses from the first stage (*S*). Simon’s minimax design is calculated as (*n*_1_,*n*,*r*_1_,*r*)=(26,45,15,32) with the MSS=45 and the ESS _0_=35.90. The minimax-EF design is found to be ((*r*_1_,*r*_2_)/*n*_1_,*r*/*n*)=((15,20)/25,32/45) with 25 patients enrolled in the first stage, and a possible total sample size of 25. The trial will be stopped for futitlity when *S*≤15 or efficacy when *S*>20 out of *n*_1_=25 patients in the first stage. The ESS_0_ is 33.3 for this design. For the proposed minimax adaptive design, the second stage sample size is allowed to change as a function of the first stage responders, and the relationship is monotonic. The first stage sample size is *n*_1_=23 and the maximum sample size is 45 and this maxmum sample size only occurs for the cases with *S*=15 and 16 responders observed from the first stage. The trial is terminated at the end of the first stage for futility or efficacy for *S*≤14 or *S*≥23, respectively. In such cases, the MSS is the first stage sample size, which is 23. When the first stage response is between 15 and 22, the corresponding second stage sample size *n*_2_(*S*) is presented in Table 3. As compared to the other designs, the adaptive minimax design has the smallest expected sample size and the smallest first stage sample size in this particular example. In the proposed design, the second stage sample size is a non-increasing function of the number of responses from the first stage, not a constant as in Simon’s design and the Minimax-EF design. It can be seen that although the adaptive optimal design has the smallest expected sample size as compared to others, the MSS of the adaptive optimal design is often much larger than that of the proposed adaptive minimax.

**Table 3:** Proposed optimal adaptive designs for *π*_*a*_=*π*_*u*_+0.2 at *α*=0.05. Simon’s minimax design (*r*_1_/*n*_1_,*r*/*n*), and the minimax that stops for futility and efficacy ((*r*_1_,*r*_2_)/*n*_1_,*r*/*n*), are provided as reference

Power = 80 %				Power = 90 %			
*S*	*n*_2_(*S*)	*n*(*S*)	*r*(*S*)	*S*	*n*_2_(*S*)	*n*(*S*)	*r*(*S*)
*π*_*u*_=0.2							
Simon: (4/18,10/33)				Simon: (5/24,13/45)			
Minimax-EF: ((2,6)/15,10/32)				Minimax-EF: ((4,9)/25,13/44)			
New: *n*_1_=19				New: *n*_1_=23			
≤4	0	19	0	≤4	0	23	0
5	13	32	10	5	21	44	12
6	13	32	10	6	21	44	13
7	13	32	9	7	21	44	13
8	13	32	10	8	21	44	13
9	11	30	10	9	21	44	13
≥10	0	19	0	10	15	38	11
				≥11	0	23	0
*π*_*u*_=0.3							
Simon: (6/19,16/39)				Simon: (7/24,21/53)			
Minimax-EF: ((8,13)/27,15/36)				Minimax-EF: ((11,17)/37,20/50)			
New: *n*_1_=20				New: *n*_1_=32			
≤5	0	20	0	≤9	0	32	0
6	16	36	14	10	18	50	19
7	16	36	15	11	18	50	20
8	16	36	15	12	18	50	20
9	16	36	15	13	18	50	20
10	16	36	15	14	18	50	20
11	16	36	15	15	18	50	20
12	14	34	15	16	18	50	20
≥13	0	20	0	17	11	43	18
				≥18	0	32	0
*π*_*u*_=0.4							
Simon: (17/34,20/39)				Simon: (12/29,27/54)			
Minimax-EF: ((17,19)/34,20/39)				Minimax-EF: ((12,19)/29,27/54)			
New: *n*_1_=16				New: *n*_1_=35			
≤6	0	16	0	≤14	0	35	0
7	23	39	20	15	18	53	26
8	23	39	20	16	18	53	27
9	23	39	20	17	18	53	27
10	23	39	20	18	18	53	27
11	23	39	21	19	18	53	26
12	22	38	20	20	17	52	26
13	16	32	18	21	17	52	26
14	9	25	16	22	17	52	26
15	5	21	15	23	17	52	27
16	3	19	16	≥24	0	35	0
*π*_*u*_=0.5							
Simon: (12/23,23/37)				Simon: (14/27,32/53)			
Minimax-EF: ((10,15)/20,23/37)				Minimax-EF: ((17,23)/34,31/51)			
New: *n*_1_=20				New: *n*_1_=28			
≤10	0	20	0	≤14	0	28	0
11	17	37	23	15	23	51	30
12	17	37	23	16	23	51	31
13	17	37	23	17	23	51	31
14	17	37	23	18	23	51	31
15	15	35	22	19	23	51	31
≥16	0	20	0	20	23	51	31
				21	21	49	29
				22	6	34	22
				≥23	0	28	0
*π*_*u*_=0.6							
Simon: (8/13,25/35)				Simon: (15/26,32/45)			
Minimax-EF: ((10,14)/17,24/33)				Minimax-EF: ((15,20)/25,32/45)			
New: *n*_1_=15				New: *n*_1_=23			
≤9	0	15	0	≤14	0	23	0
10	18	33	24	15	22	45	32
11	18	33	24	16	22	45	32
12	17	32	23	17	21	44	31
13	16	31	22	18	21	44	31
14	14	29	21	19	21	44	31
15	14	29	21	20	10	33	24
				21	10	33	25
				22	8	31	24
				≥23	0	23	0
*π*_*u*_=0.7							
Simon: (19/23,21/26)				Simon: (13/18,26/32)			
Minimax-EF: ((19,20)/23,21/26)				Minimax-EF: ((13,18)/18,26/32)			
New: *n*_1_=13				New: *n*_1_=18			
≤9	0	13	0	≤13	0	18	0
10	12	25	21	14	14	32	26
11	12	25	21	15	14	32	26
12	12	25	20	16	14	32	26
13	7	20	16	17	14	32	26
				≥17	3	21	18

**Table 4:** Proposed optimal adaptive designs for *π*_*a*_=*π*_*u*_+0.15 at *α*=0.05. Simon’s minimax design (*r*_1_/*n*_1_,*r*/*n*), and the minimax that stops for futility and efficacy ((*r*_1_,*r*_2_)/*n*_1_,*r*/*n*), are provided as reference

Power=80 %				Power=90 %			
*S*	*n*_2_(*S*)	*n*(*S*)	*r*(*S*)	*S*	*n*_2_(*S*)	*n*(*S*)	*r*(*S*)
*π*_*u*_=0.1							
Simon: (2/22,7/40)				Simon: (3/31,9/55)			
Minimax-EF: ((4,6)/33,7/38)				Minimax-EF: ((6,8)/47,9/53)			
New: *n*_1_=18				New: *n*_1_=33			
≤1	0	18	0	≤3	0	33	0
2	20	38	7	4	20	53	8
3	20	38	7	5	20	53	9
4	19	37	6	6	20	53	9
5	19	37	6	7	18	51	8
6	18	36	6	8	17	50	8
≥7	0	18	0	≥9	0	33	0
*π*_*u*_=0.2							
Simon: (6/31,15/53)				Simon: (8/42,21/77)			
Minimax-EF: ((6,13)/31,15/53)				Minimax-EF: ((13,18)/62,21/76)			
New: *n*_1_=31				New: *n*_1_=47			
≤6	0	31	0	≤9	0	47	0
7	22	53	15	10	27	74	20
8	22	53	15	11	27	74	20
9	22	53	15	12	27	74	20
10	22	53	15	13	27	74	20
11	22	53	15	14	26	73	20
12	21	52	15	15	26	73	20
≥13	0	31	0	16	26	73	20
				17	25	72	20
				18	14	61	18
				≥19	0	47	0
*π*_*u*_=0.3							
Simon: (16/46,25/65)				Simon: (27/77,33/88)			
Minimax-EF: ((13,19)/43,25/64)				Minimax-EF: ((27,33)/77,33/88)			
New: *n*_1_=32				New: *n*_1_=51			
≤9	0	32	0	≤15	0	51	0
10	32	64	24	16	37	88	33
11	32	64	25	17	37	88	33
12	32	64	25	18	37	88	33
13	32	64	25	19	37	88	33
14	32	64	25	20	37	88	33
15	31	63	24	21	37	88	33
16	30	62	24	22	37	88	33
17	29	61	24	23	37	88	33
18	24	56	22	24	37	88	34
≥19	0	32	0	25	36	87	33
				26	34	85	33
				27	34	85	33
				≥28	0	51	0
*π*_*u*_=0.4							
Simon: (28/59,34/70)				Simon: (24/62,45/94)			
Minimax-EF: ((16,23)/41,34/69)				Minimax-EF: ((21,31)/55,45/94)			
New: *n*_1_=37				New: *n*_1_=52			
≤15	0	37	0	≤20	0	52	0
16	32	69	33	21	42	94	44
17	32	69	34	22	42	94	45
18	32	69	34	23	42	94	45
19	32	69	34	24	42	94	45
20	32	69	34	25	42	94	45
21	31	68	33	26	42	94	45
22	31	68	33	27	42	94	45
23	31	68	33	28	42	94	45
24	21	58	29	29	42	94	45
≥25	0	37	0	30	42	94	45
				31	39	91	43
				≥32	0	52	0
*π*_*u*_=0.5							
Simon: (39/66,40/68)				Simon: (28/57,54/93)			
Minimax-EF: ((39,40)/66,40/68)				Minimax-EF: ((30,38)/59,54/93)			
New: *n*_1_=54				New: *n*_1_=55			
≤28	0	54	0	≤28	0	55	0
29	13	67	39	29	38	93	54
30	13	67	40	30	38	93	54
31	13	67	40	31	38	93	54
32	13	67	40	32	38	93	54
33	13	67	40	33	38	93	54
34	13	67	39	34	38	93	54
35	13	67	39	35	38	93	53
36	13	67	39	36	38	93	54
37	9	63	38	37	38	93	53
≥38	0	54	0	≥38	0	55	0
*π*_*u*_=0.6							
Simon: (18/30,43/62)				Simon: (48/72,57/84)			
Minimax-EF: ((16,22)/27,43/62)				Minimax-EF: ((48,53)/72,57/84)			
New: *n*_1_=32				New: *n*_1_=58			
≤19	0	32	0	≤37	0	58	0
20	29	61	42	38	26	84	57
21	29	61	42	39	26	84	57
22	29	61	42	40	26	84	57
23	28	60	42	41	26	84	57
24	28	60	42	42	25	83	57
25	28	60	42	43	25	83	57
26	27	59	41	44	23	81	56
27	27	59	41	≥45	0	58	0
28	15	47	34				
≥29	0	32	0				
*π*_*u*_=0.7							
Simon: (16/23,39/49)				Simon: (33/44,53/68)			
Minimax-EF: ((16,21)/23,39/49)				Minimax-EF: ((29,35)/41,51/65)			
New: *n*_1_=25				New: *n*_1_=37			
≤18	0	25	0	≤26	0	37	0
19	24	49	39	27	28	65	51
20	24	49	39	28	28	65	51
21	24	49	39	29	28	65	51
22	23	48	38	30	28	65	51
23	8	33	26	31	27	64	50
≥24	0	25	0	32	27	64	50
				33	24	61	48
				≥34	0	37	0

As suggested by one of the reviewers, we compare the probability of early termination (PET) at the first stage for these designs. The PET is defined as the probability of a study that is stopped at the first stage due to either futility or efficacy. We present the PET of the three designs with *π*_*a*_=*π*_*u*_+0.2 and 80 % power in Table 5. The PET of the new adaptive design is always less than that by Simon’s minimax design in these cases. There is no clear relationship between the minimax-EF design and the new design with regard to the PET. It can be seen that the PET of the new design is more consistent as compared to the competitors.

**Table 5:** Probability of early termination at the first stage for the designs with *π*_*a*_=*π*_*u*_+0.2 and 80 % power

*π*_*u*_	Simon	Minimax-EF	New adaptive design
0.2	0.716	0.402	0.674
0.3	0.666	0.582	0.417
0.4	0.913	0.921	0.527
0.5	0.661	0.589	0.589
0.6	0.647	0.554	0.597
0.7	0.946	0.949	0.579

**Fig. 2:**
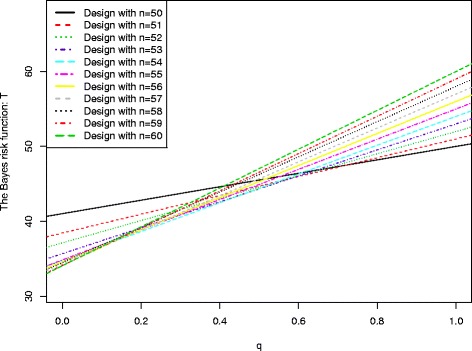
The Bayes risk function as a function of the weight value *q* in searching for an admissible adaptive design for (*α*,*β*,*π*_*u*_,*π*_*a*_)=(0.05,0.1,0.3,0.5)

Figure 2 displays the lines of the Bayes risk function *T* as a function of *q* for each design to attain 90 % power at the significance level of 0.05, with *π*_*u*_=0.3 and *π*_*a*_=0.5. The minimax adaptive design and the optimal adaptive design are presented in Table 3, with *n*_*min*_=50 and *n*_*opt*_=60. Therefore, a total of 11 lines are displayed in the figure to represent the optimal designs when *n* is between 50 and 60. In order to identify an admissible design for a given range of *q*, one has to first compute the intersections among these 11 lines, and the maximum number of intersections between 0 and 1 is $\binom {11}{2}=55$. Within these 55 intersections, 2 of them are out of the range of [0,1], this leads to a total of 53 intersections between 0 and 1. After sorting the x-values of these intersections, the design among these 11 designs is the admissible design for a given range of *q* when this design has the smallest *T* over this range, see Table 6. It can be seen that the optimal adaptive design is the admissible design when *q* is close to 0, and the minimax design is the admissible design when *q* is close to 1.

**Table 6:** Admissible adaptive designs for (*α*,*β*,*π*_*u*_,*π*_*a*_)=(0.05,0.1,0.3,0.5)

Interval of *q*	n	ESS_0_	Comment
[0.000,0.040]	60	34.08	Optimal design
[0.040,0.105]	59	34.12	
[0.105,0.132]	57	34.36	
[0.132,0.468]	54	34.81	
[0.468,0.580]	53	35.69	
[0.580,0.721]	51	38.45	
[0.721,1.000]	50	41.03	Minimax design

### Application

**Fig. 3:**
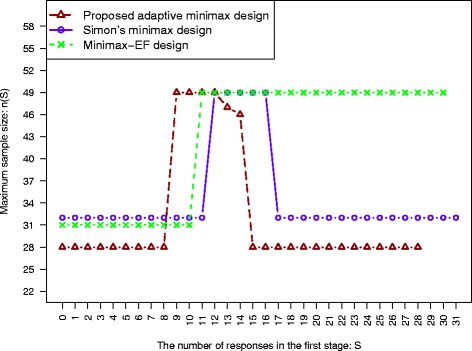
For the urothelial cancer trial with parameters (*α*,*β*,*π*_*u*_,*π*_*a*_)=(0.1,0.2,0.35,0.5), the design parameters for Simon’s minimax design, the minimax-EF design and the proposed adaptive minimax design. The maximum sample size, *n*(*S*), is plotted as a function of the number of responses from the first stage, *S*

We revisit the urothelial cancer trial with the neoadjuvant therapy [10]. Simon’s minimax design was used for study design to attain 80 % power at the significance level of *α*=0.1. The research team expected a 15 % increase in response rate as compared to the priori estimated response rate *π*_*u*_=35 %. The design parameters using Simon’s minimax design are: (*n*_1_,*n*,*r*_1_,*r*)=(31,49,10,21) with the ESS_0_=40.8. The minimax-EF design is: ((*r*_1_,*r*_2_)/*n*_1_,*r*/*n*)=((11,16)/32,21/49). The design parameters, (*n*_1_,*n*(*S*),*r*(*S*)), for the proposed adaptive minimax design are presented in Table 1, and also plotted in Fig. 3. They all have the same maximum sample size 49, but the expected sample size under the null for the proposed design is smaller, 38.9 VS 40.8(Simon’s design), and 39.2 (the minimax-EF design). The adaptive design is also flexible to allow the second stage sample size and its associted critical value to depend on the result from the first stage.

## Discussion and conclusions

We develop a new minimax adaptive two-stage design for use in phase II clinical trials to assess the new treatment’s activity. The software program to implement the adaptive designs in this article is written in the statistical language, R [11–14], and it is available per request from the first author (guogen.shan@unlv.edu) or the corresponding author (jtao@263.net). We are also working together to develop a new R package to implement the adaptive minimax and admissible designs from this article and the adaptive optimal design by Shan et al. [8]. The proposed design allows the second stage sample size and its associated critical value to depend on the result from the first stage. The proposed design satisfies the monotonicity property of the relationship between the second stage sample size and the first stage responders, which is an important feature for a practical application.

The MSS of the proposed adaptive minimax design is always less than or equal to that of the minimax-EF design. We consider this as an important advantage of the minimax adaptive design to reduce the computational intensity as compared to adaptive designs based on the optimal criteria [8], where the upper bound of the sample size has to be set in the design search process. To reduce the computational time, one may use a backward search method as in this article, starting with the maximum sample size from the minimax-EF design. In addition, when the proposed design and other designs have the same MSS, the expected sample size under the null of the proposed design is always smaller than others.

The proposed adaptive design assumes a monotonic relationship between the second stage sample size and the first stage result. In practice, an investigator may want to accrue more patients in the second stage when the number of response from the first stage is large, to obtain as much information as possible from the clinical study. In this case, an additional constraint can be added during the design search to meet the investigator’s requirement: the second stage sample sizes are the same when *S* is above *S*_*c*_, where *S*_*c*_ can be determined by the new constraint from the investigator. The new constraint added in the design search should be clinically meaningful.

The naive point estimate for the probability of response rate is calculated as the number of responses divided by the total number of patients, and it is well known that this estimate is biased. In the traditional Simon’s design, Jung and Kim [15] derived the uniformly minimum variance unbiased estimate for the probability of response based on the Rao-Blackwell theorem. To the best of our knowledge, no unbiased estimate for the probability of response has been proposed in an adaptive two-stage design setting. This may be due to the complexity of an adaptive design as compared to the traditional sample size fixed design.

Randomized clinical trials are used in clinical trials by comparing the new treatment or therapy to the best available treatment for disease. Randomized clinical trials are preferable in the majority of studies to reduce the selection bias and confounding effects, thus capturing the true effectiveness of the new treatment. The widely used two-stage design for a two-arm study with binary outcomes is the one due to Thall et al. [16], that does not allow the second stage sample size to change from the results of the first stage. We will extend the adaptive approach from the one-arm study to this two-arm study to develop a new adaptive two-stage design for a randomized clinical trial with dichotomous endpoints.

## Abbreviations

ESS_0_, the expected sample size under the null; MMS, maximum sample size
